# Radiosynthesis and Evaluation of 11C-Labeled Isoindolone-Based Positive Allosteric Modulators for Positron Emission Tomography Imaging of Metabotropic Glutamate Receptor 2

**DOI:** 10.1021/acsptsci.4c00261

**Published:** 2024-07-10

**Authors:** Yinlong Li, Kenneth Dahl, Peter Johnström, Katarina Varnäs, Lars Farde, Christer Halldin, Amy Medd, Donna Maier, Mark E. Powell, Jiahui Chen, Richard Van, Jimmy Patel, Ahmad Chaudhary, Yabiao Gao, Zhendong Song, Achi Haider, Yihan Shao, Charles S. Elmore, Steven Liang, Magnus Schou

**Affiliations:** † Department of Radiology and Imaging Sciences, Emory University, 1364 Clifton Road, Atlanta, Georgia 30322, United States; ‡ PET Science Centre, Precision Medicine and Biosamples, Oncology R&D, AstraZeneca, Karolinska Institutet, Stockholm S-17176, Sweden; § Department of Clinical Neuroscience, Centre for Psychiatry Research, Karolinska Institutet and Stockholm County Council, Stockholm S-17176, Sweden; ∥ Neuroscience, BioPharmaceuticals R&D, AstraZeneca, Wilmington, Delaware 19803, United States; ⊥ Early Chemical Development, Pharmaceutical Sciences, R&D, AstraZeneca Pharmaceuticals, Gothenburg 43183, Sweden; # Department of Chemistry and Biochemistry, University of Oklahoma, Norman, Oklahoma 73019-5251, United States

**Keywords:** mGluR_2_, positive allosteric modulator, AZ12559322, positron emission tomography, radioligand

## Abstract

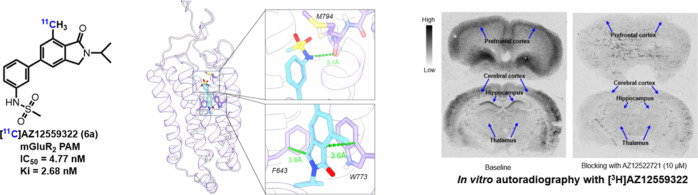

The metabotropic glutamate receptor 2 (mGluR_2_) has emerged as a potential therapeutic target for the treatment of various neurological diseases, prompting substantial interest in the development of mGluR_2_-targeted drug candidates. As part of our medicinal chemistry program, we synthesized a series of isoindolone derivatives and assessed their potential as mGluR_2_ positive allosteric modulators (PAMs). Notably, AZ12559322 exhibited high affinity (*K*_i_ mGluR_2_ = 1.31 nM) and an excellent in vitro binding specificity of 89% while demonstrating selectivity over other mGluR subtypes (>4000-fold). Autoradiography with the radiolabeled counterpart, [^3^H]AZ12559322, revealed a heterogeneous accumulation with the highest binding in mGluR_2_-rich brain regions. Radioligand binding was significantly reduced by pretreatment with nonradioactive mGluR_2_ PAMs in brains of rats and nonhuman primates. Although positron emission tomography imaging of [^11^C]AZ12559322 (**6a**) revealed low brain uptake in a nonhuman primate, this study provides valuable guidance to further design novel isoindolone-based mGluR_2_ PAMs with improved brain exposure.

Glutamate constitutes the primary excitatory neurotransmitter within the central nervous system (CNS) and plays a vital role in synaptic transmission and neuroplasticity via activation of ionotropic (iGluRs) and metabotropic glutamatergic receptors (mGluRs).^1−4^ Of note, mGluRs belong to the superfamily of G-protein-coupled receptors (GPCRs) and are categorized in three distinct subtypes: group I (mGluR_1_ and mGluR_5_), group II (mGluR_2_ and mGluR_3_), and group III (mGluRs 4, 6, 7, and 8). Notably, mGluR_2_ and mGluR_3_ are preferentially localized on presynaptic nerve terminals to regulate glutamatergic synaptic transmission and ion channels via coupling to *G*_i/o_ signaling.^5−7^

The subtype mGluR_2_ is highly enriched in several brain regions including the cerebral cortex, caudate-putamen, and hippocampus. Under physiological conditions, mGluR_2_ negatively modulates the release of glutamate and GABA. While dysregulation of mGluR_2_ has been implicated in neurodegenerative and neuropsychiatric disorders, restoring mGluR_2_ function has been suggested as a potential therapeutic concept, particularly in conditions involving excessive glutamate transmission.^8,9^ Indeed, various studies have linked mGluR_2_ expression and function to the pathophysiology of schizophrenia, depression, Alzheimer’s disease (AD), and Parkinson’s disease (PD).^10−13^ For instance, altered levels of mGluR_2_ have been observed in the prefrontal cortex (PFC) of animal models of depression and patients with depression, suggesting a therapeutic potential of mGluR_2_ ligands in this patient population.^14,15^ Indeed, both animal studies and clinical trials support that mGluR_2_ agonists exert anxiolytic and antipsychotic activity.^16−18^ For instance, LY2140023, a prodrug and mGluR_2/3_ agonist developed by Eli Lilly, achieved success in a phase II study.^19−21^

Of note, there is an allosteric modulator-binding pocket in the seven transmembrane domain (7TM) of mGluRs. This binding site provides a promising pharmacological strategy to develop selective ligands that modulate mGluR_2_ through allosteric mechanisms.^22,23^ The latter has channeled the development of positive allosteric modulators (PAMs)^24,25^ and negative allosteric modulators (NAMs)^26,27^ that have demonstrated effective modulation of mGluR_2_ and exhibited improved subtype selectivity as well as blood–brain barrier (BBB) penetration.^22,23^

Positron emission tomography (PET) is a noninvasive in vivo imaging technique that enables the visualization and quantification of neuroreceptor binding.^28−30^ Given the growing interest in mGluR_2_-targeted therapeutics for neurological and psychiatric disorders, the availability of a suitable mGluR_2_ PET ligand would substantially facilitate the clinical development of potential drug candidates.^31,32^ To date, several mGluR_2_ PET ligands have been reported, including both PAM- and NAM-based probes (Figure 1). Among the PAMs, [^11^C]JNJ-42491293 has been translated to humans; however, it exhibited significant off-target binding.^33−35^ The benzimidazole derivative, [^11^C]mG2P001, has been evaluated in nonhuman primates (NHPs); however, pretreatment with nonradiolabeled mG2P001 led to an increase of [^11^C]mG2P001 brain exposure.^36,37^ Similarly, other triazolopyridine-based radioligands such as [^18^F]JNJ-46356479^38^ and [^18^F]mG2P026^39^ also exhibited enhanced brain exposure after pretreatment with unlabeled PAMs in rats and NHPs. In terms of PET ligands derived from NAMs, [^11^C]QCA,^40^ [^11^C]MG2–1904,^41^ [^11^C]MK-8056,^42^ and [^11^C]mG2N001 have been developed as radioligands but reported to have limited BBB permeability.^43^ In summary, due to the lack of a clinically validated mGluR_2_ PET ligand, there is an unmet need for the extended development of specific and selective mGluR_2_ radioligands.

**Figure 1:**
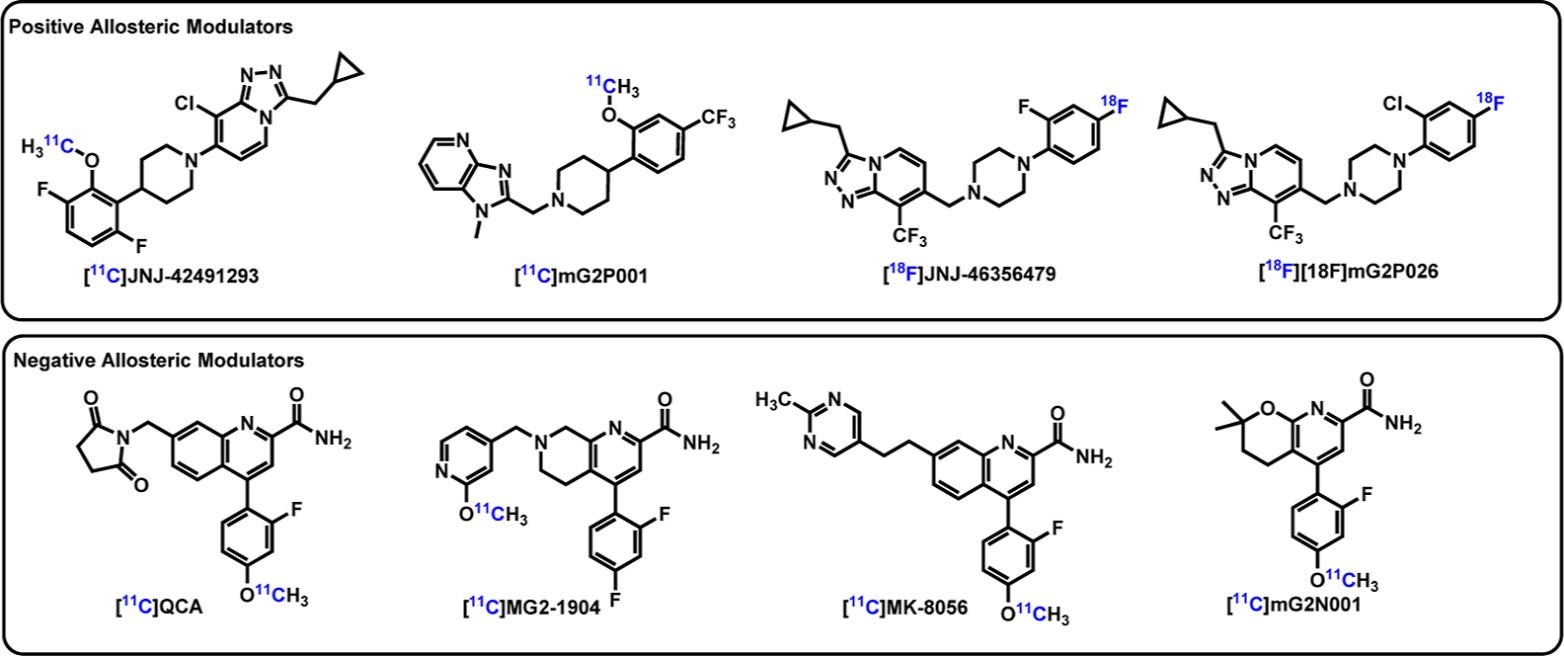
Reported mGluR_2_ PET ligands.

With this in mind, we herein report the synthesis and ^11^C-labeling of three isoindolone analogues as potential mGluR_2_ PAM PET ligands. The chemistry was followed by in vitro autoradiography (ARG) and in vivo PET imaging of binding in NHP (Figure 2).

**Figure 2:**
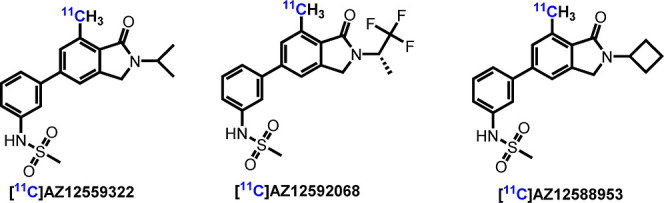
Chemical structures of novel mGluR_2_ PAM PET ligands.

## Results and Discussion

### Chemistry

**Scheme 1:**
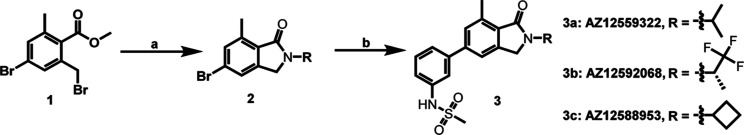
Syntheses of Reference Compounds **3a**–**3c** Reagents and conditions: (a) NH_2_R, K_2_CO_3_, H_3_BO_3_, acetonitrile, rt to reflux, overnight; (b) 3-(methylsulfonylamino)phenylboronic acid, Pd(PPh_3_)_2_Cl_2_, Cs_2_CO_3_, DME/H_2_O/EtOH (7:3:2).

Structurally, compounds AZ12559322 (**3a**), AZ12592068 (**3b**), and AZ12588953 (**3c**) feature a 7-methyl isoindolone skeleton, and their syntheses are illustrated in Scheme 1. Briefly, the isoindolone ring **2** was assembled from methyl 4-bromo-2-(bromomethyl)-6-methylbenzoate **1** with different alkylamines. Subsequently, Suzuki coupling reaction of **2** with 3-(methylsulfonylamino)phenylboronic acid afforded **3a**–**3c** with distinct alkyl substituents. All three compounds were obtained in high purity over two steps.

### Pharmacology

**Figure 3:**
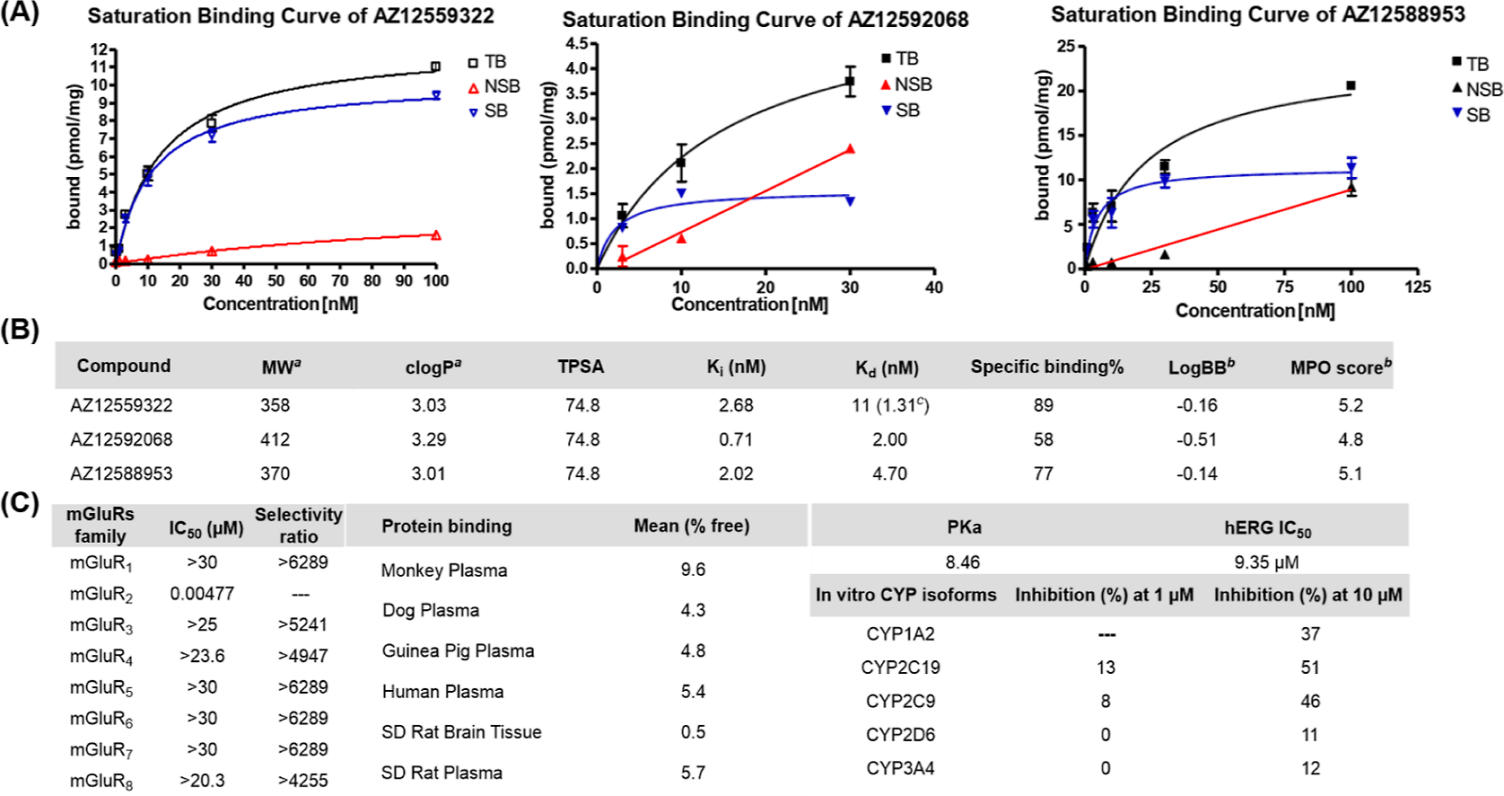
(A) Saturation binding curves for [^3^H]AZ12559322, [^3^H]AZ12592068, and [^3^H]AZ12588953. (B) Pharmacology and physiochemical properties of unlabeled AZ12559322, AZ12592068, and AZ12588953. (C) Selectivity toward mGluR family receptors, p*K*_a_, hERG channel, and metabolic stability data of AZ12559322. ^a^Values were calculated with ChemDraw 21.0 software. ^b^Values were predicted with ACD/laboratories. ^c^Determined by in vitro ARG of [^3^H]AZ12559322 on rat brain tissues (see Figure S1 in the Supporting Information).

The pharmacology and physicochemical properties of AZ12559322 (**3a**), AZ12592068 (**3b**), and AZ12588953 (**3c**) are summarized in Figure 3. To determine the binding affinities, saturation binding experiments of the corresponding tritiated ligands were conducted on CHO membranes stably expressing mGluR_2_. As shown in Figure 3A, total binding (TB), nonspecific binding (NSB), and specific binding (SB) of [^3^H]AZ12559322, [^3^H]AZ12592068, and [^3^H]AZ12588953 were measured at different concentrations. Compounds **3a**–**3c** exhibited high binding affinity to mGluR_2_ with low *K*_i_ (0.71–2.68 nM) and *K*_d_ values (2.0–11 nM). The *K*_d_ value of **3a** was calculated as 1.31 nM based on the binding data of in vitro ARG of [^3^H]AZ12559322 on rat brain tissues (see Figure S1 in the Supporting Information). Notably, AZ12559322 displayed relatively high specific binding to mGluR_2_ in vitro (89% of SB). The three compounds had similar *c* Log *P* values (3.01 to 3.29) and identical topological polar surface areas (TPSAs) of 74.8. The blood–brain barrier permeability (log BB) values of **3a**–**3c** were predicted in the range of −0.14 to −0.51. Multiparameter optimization (MPO) is a valuable tool to evaluate the BBB permeability of drug candidates. In general, CNS MPO scores on a scale between 0 and 6, and the score value ≥4.0 is highly desirable. AZ12559322 (**3a**) showed the highest CNS MPO score of 5.2, indicating its significant potential as a candidate (Figure 3B).^44^ Therefore, AZ12559322 was selected as the most promising mGluR_2_ PAM for further ADME profiling. To investigate the selectivity of AZ12559322 for mGluR_2_, further counter-screening of AZ12559322 was conducted, and the results are detailed in Figure 3C. Of note, AZ12559322 showed high mGluR_2_ activity (IC_50_ = 4.7 nM) and excellent selectivity (>4000-fold) against all other mGluRs including the structurally related Group II mGluR_3_ (IC_50_ > 25 μM). The plasma protein binding (PPB) study indicated that AZ12559322 possesses reasonable unbound fractions in the plasma of various species (e.g., cynomolgus monkey, dog, and guinea pig). In addition, AZ12559322 exhibited a reasonable acidity value (p*K*_a_ = 8.46), a hERG channel IC_50_ of 9.35 μM, and no relevant interactions with metabolic cytochrome P450 (CYP) isoforms.

### Molecular Docking

**Figure 4:**
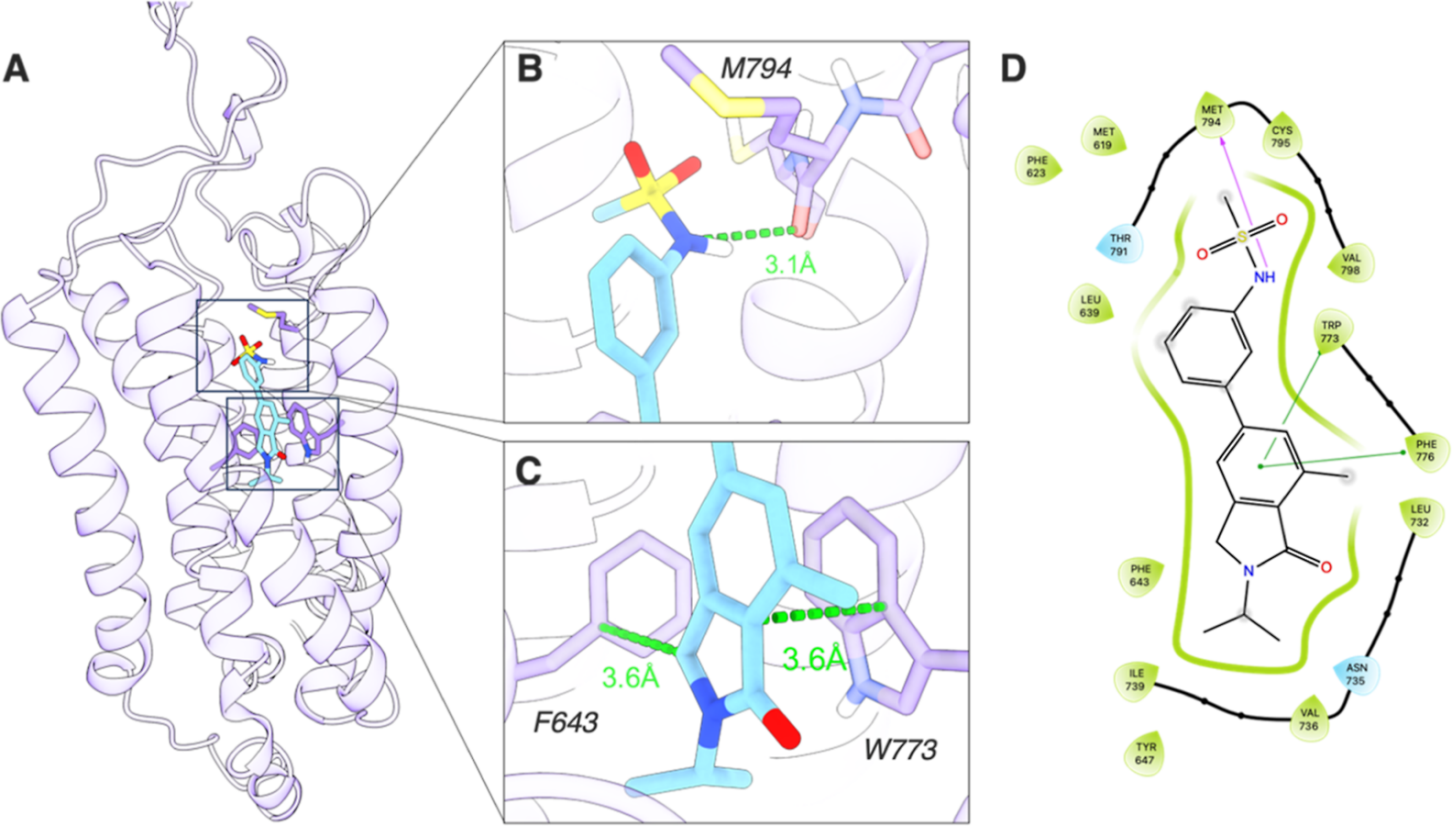
(A) Overview of AZ12559322 (blue) binding pose with a mGluR_2_ homology model (purple). (B) Distance of hydrogen bond interaction with M794 (green dashed line). (C) pi–pi interactions with residues F643 and W773. Green dashed lines highlight the nearest carbon–carbon distance. (D) 2D interaction plots of binding orientation. The pocket highlight in green indicates a hydrophobic environment, green lines are pi–pi interactions, and the purple arrow is the hydrogen bond interaction.

To investigate the protein–ligand interactions, AZ12559322 was docked into a mGluR_2_ homology model developed by Yuan and co-workers.^39^ Like previous PAM ligands report, the binding pocket of AZ12559322 was found to be buried in the α-helical transmembrane region of mGluR_2_ (Figure 4A). The favorable binding orientation is supported by hydrogen bond interactions with residues, M794, and pi–pi interactions with residues, W643 and W773. Toward the solvent-exposed region, the NH group of the ligand formed a hydrogen bond with the carbonyl oxygen of M794 with the N–O distance measured at 3.1 Å (Figure 4B). Additionally, the indole moiety forms pi–pi interactions with two residues, F643 and W773, at a distance of 3.6 Å from both side chains (Figure 4C). This orientation enables the indole group to be buried within a hydrophobic pocket composed of residues L639, F643, L732, V736, I739, and V798. These findings suggested that AZ12559322 possesses potentially high binding affinity to mGluR_2_.

### In Vitro Autoradiography

**Figure 5:**
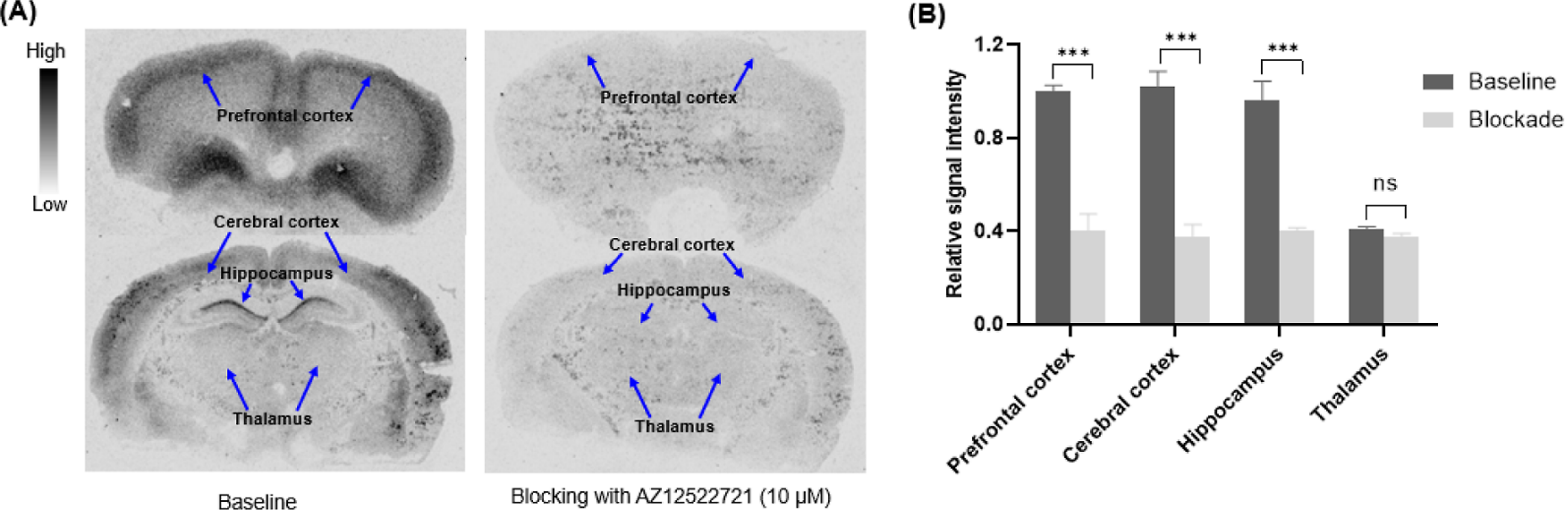
In vitro autoradiography of rat brain tissue with [^3^H]AZ12559322. (A) Autoradiograms after incubation with either [^3^H]AZ12559322 only (baseline) or 10 μM AZ12522721 (blockade). (B) Quantitative analyses of the autoradiograms under baseline and blocking conditions (*n* = 4). ***p ≤ 0.001, ns = nonsignificant.

**Figure 6:**
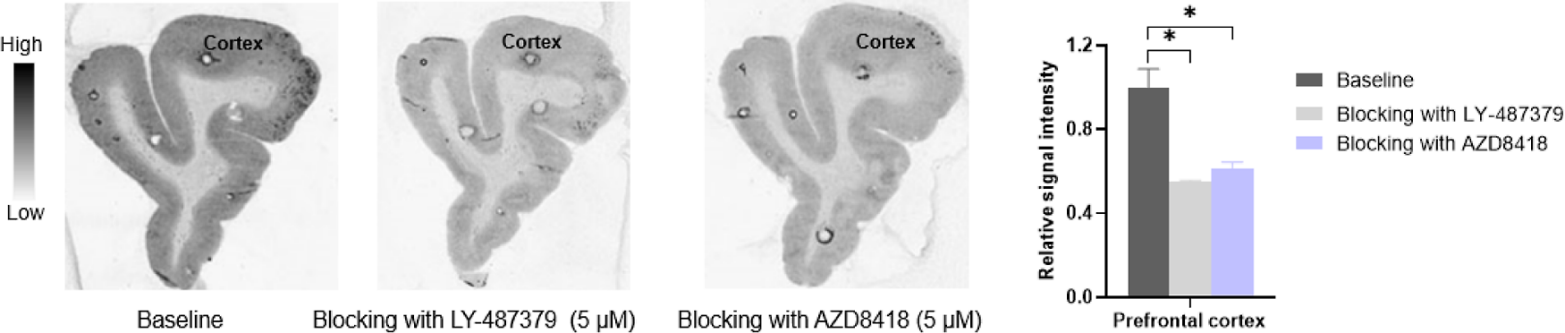
In vitro autoradiography and quantitative analyses of NHP prefrontal cortex with [^3^H]AZ12559322 (*n* = 2). Total binding and nonspecific binding in the presence of 5 μM of LY-487379 or AZD8418. *p < 0.05.

Encouraged by the promising ligand–protein binding ability of AZ12559322, in vitro autoradiography (ARG) studies were conducted on rat brain tissues to assess the binding specificity of [^3^H]AZ12559322. Representative autoradiograms and quantitative analysis are shown in Figure 5A,B, respectively. Consistent with previous reports of the regional mGluR_2_ expression in the rat brain,^45^ [^3^H]AZ12559322 showed heterogeneous distribution with high levels in the prefrontal cortex, cerebral cortex, and hippocampus, whereas relatively low binding was detected in the thalamus (Figure 5A). Co-incubation with a high concentration of the mGluR_2_ PAM AZ12522721 (10 μM) led to a marked reduction of the signal intensity in all mGluR2 rich regions, indicating high specific binding in vitro (Figure 5B). Subsequently, in vitro ARG studies with brain regions from cynomolgus monkeys were conducted to confirm the specificity in higher species. As depicted in Figure 6, blocking studies with the reference mGluR_2_ PAM LY-487379 (5 μM) and AZD8418 (5 μM) resulted in diminished binding (ca. 40–45%) in the prefrontal cortex of the NHP brain. Furthermore, in situ GTPγS ARG studies of an isoindolone analogue AZD8529 in the rat and NHP brain tissues confirmed the allosteric binding mechanism and positive modulation of this compound series in the presence of a known agonist, LY379268.^46^ Subsequent immunohistochemical analyses verified that AZD8529 was binding to the regions with high expression of mGluR_2_ (see Figure S2 in the Supporting Information). Altogether, these studies indicated high mGluR_2_ in vitro binding specificity of AZ12559322 on rat and NHP brain tissue sections and positive allosteric mechanism of action.

### Radiochemistry and PET Imaging Studies in NHPs

**Scheme 2:**
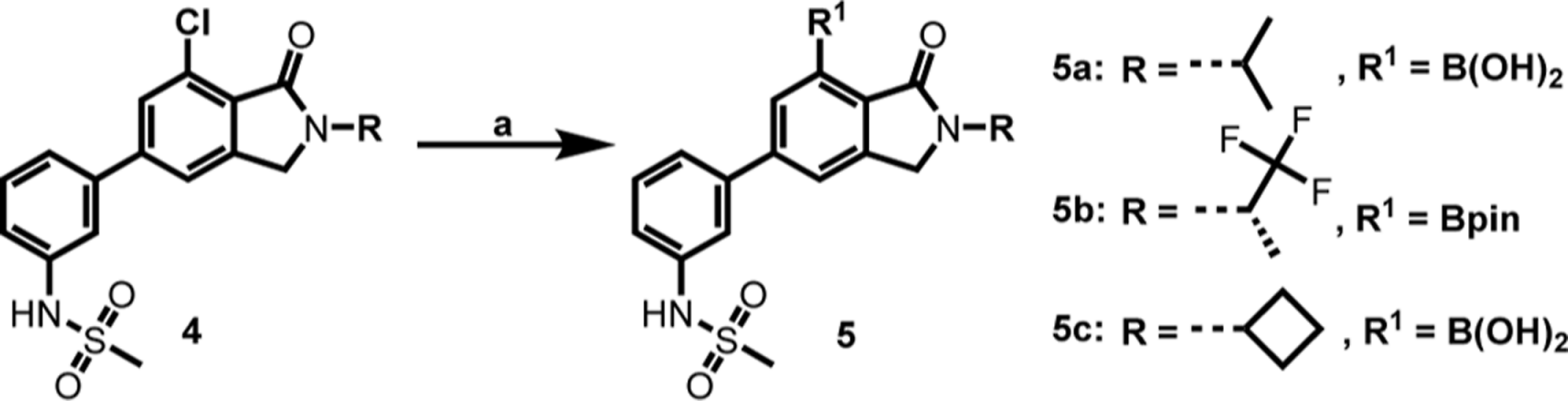
Preparation of Precursors **5** for Radiolabeling Reagents and conditions: (a) Pd_2_(dba)_3_, XPhos, KOAc, bis(pinacolato)diboron, dioxane, 110 °C, 1–4 h. **5a**: 28% yield, **5b**: 10% yield, **5c**: 24% yield.

**Scheme 3:**
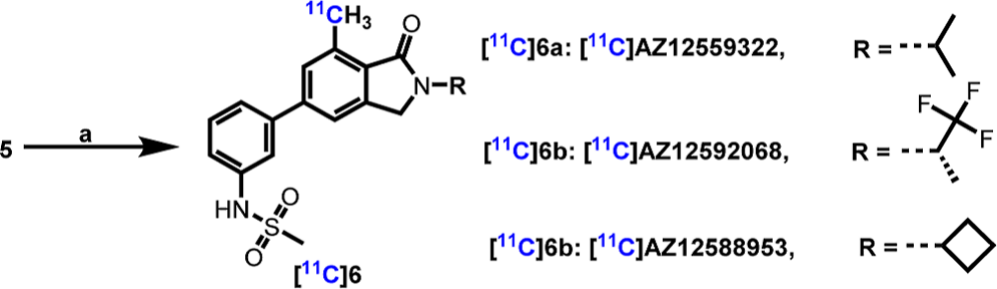
Radiosynthesis of [^11^C]AZ12559322, [^11^C]AZ12592068, and [^11^C]AZ12588953 Reagents and conditions: (a) [^11^C]CH_3_I, Pd(dppf)Cl_2_, K_2_HPO_4_, DMF, 100 °C, 4 min, *n* = 2.

Considering that the labeling of the methyl group on the phenyl ring of **3a**–**3c** is a convenient strategy to introduce carbon-11, while maintaining pharmacological activity,^47,48^ the corresponding boronic acid/ester modified precursors **5** were prepared via Miyaura borylation of aryl halides **4** (Scheme 2). Subsequently, the radiosynthesis was accomplished by palladium-mediated cross-coupling reaction of precursors **5** and [^11^C]methyl iodide (Scheme 3). [^11^C]AZ12559322 ([^11^C]**6a**) was isolated with a radiochemical yield (RCY) of 13.4% (decay-corrected, *n* = 2) at the end of synthesis (EOS, synthesis time 45 min). [^11^C]AZ12592068 ([^11^C]**6b**) and [^11^C]AZ12588953 ([^11^C]**6c**) were obtained with decay-corrected RCYs of 17.9% (EOS, synthesis time 48 min, *n* = 2) and 14.3% (EOS, synthesis time 51 min, *n* = 2), respectively. All radioligands exhibited a molar activity (Am) exceeding 37 GBq/μmol with a radiochemical and chemical purity of >98%.

**Figure 7:**
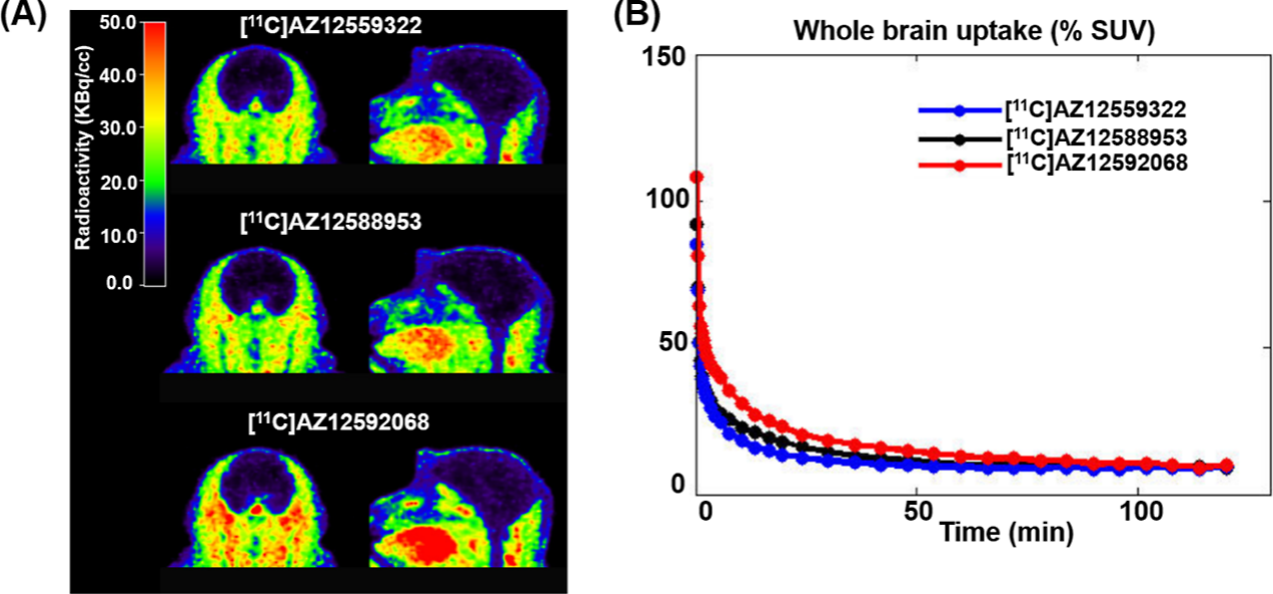
Summed (9–123 min) PET images (A) and time–activity curves (B) of [^11^C]AZ12559322, [^11^C]AZ12592068, and [^11^C]AZ12588953.

With all three radioligands in hand, we sought to evaluate the feasibility of these radioligands for quantifying mGluR_2_ PAM occupancy in the NHP brain. PET imaging studies of [^11^C]AZ12559322, [^11^C]AZ12592068, and [^11^C]AZ12588953 were performed in a cynomolgus monkey. Radioactivity was acquired for 120 min after IV injection of the respective radioligand. Despite encouraging in vitro properties, baseline studies of all three radioligands showed low brain exposure followed by rapid washout (Figure 7), thus precluding a more detailed characterization of the binding. Further structural modification of these isoindolone derivatives is necessary to enhance brain exposure and provide a suitable radioligand for in vivo imaging.

## Conclusions

We have synthesized three isoindolone derivatives as novel mGluR_2_ PAM PET radioligands. The pharmacological assessment indicated that AZ12559322 has high mGluR_2_ binding affinity (*K*_i_ = 1.37 nM) and displays excellent selectivity over other mGluR subtypes (>4000-fold). In vitro ARG studies showed that [^3^H]AZ12559322 has specific binding in mGluR_2_ high expression regions of the rat and NHP brains. The radiosyntheses of [^11^C]AZ12559322, [^11^C]AZ12592068, and [^11^C]AZ12588953 were achieved in good radiochemical yields, high purity, and molar activity. Although PET imaging in NHP revealed low brain uptake and fast washout for those ligands, this study provides valuable insights for the further development of isoindolone-based mGluR_2_ PAMs for PET imaging. The new radioligands should be designed with the aim to be devoid of efflux transport and thereby improve the BBB permeability while still maintaining its high affinity and selectivity toward the mGluR_2_ receptor. For instance, developing radioligands without the sulfonamide moiety is a particularly promising direction.

## Experimental Section

### Materials and Methods

All solvents and reagents were obtained from commercially available sources and used without further purification. *N*-(3-(7-Chloro-2-isopropyl-1-oxoisoindolin-5-yl)phenyl)methanesulfonamide **4a**, (*S*)-*N*-(3-(7-chloro-1-oxo-2-(1,1,1-trifluoropropan-2-yl)isoindolin-5-yl)phenyl)methanesulfonamide **4b**, *N*-(3-(7-chloro-2-cyclobutyl-1-oxoisoindolin-5-yl)phenyl)methanesulfonamide **4c**, *N*-[3-(2-isopropyl-7-methyl-1-oxo-2,3-dihydro-1*H*-isoindol-5-yl)phenyl]methanesulfonamide **3a** (AZ12229322), (*S*)-*N*-(3-(2-(1,1,1-trifluoropropan-2-yl)-7-methyl-1-oxoisoindolin-5-yl)phenyl)methanesulfonamide **3b** (AZ12592068), and *N*-(3-(2-cyclobutyl-7-methyl-1-oxoisoindolin-5-yl)phenyl)methanesulfonamide **3c** (AZ12588953) were obtained from AstraZeneca Medicinal Chemistry. Analytical HPLC analyses were performed using an Agilent 1100 series HPLC system with a LabLogic in-line radioactive detector using a mobile phase of MeCN in 0.1% formic acid a gradient elution of 10 to 100% over 10 min (Method A), 35 to 45% over 20 min (Method B), or 50 to 90% over 20 min (Method C) on a 4.6 × 100 mm Phenomenex Luna C18(2) column or 50 to 90% (Method D) over 20 min on a 4.6 × 100 mm Phenomenex Synergi RP-Polar followed by a 5 min wash with 100% MeCN. Semipreparative HPLC performed on a Phenomenex Luna C-18(2) column (250 × 10 mm, 5 μm) using gradient elution over 40 min with MeCN-0.1% TFA mobile phase at 3 mL/min. Preparative HPLC purifications were performed on a Phenomenex Luna C-18(2) column (250 × 21.2 mm, 5 μm) using gradient elution with MeCN-0.1% TFA mobile phase at 15 mL/min. Silica gel purifications were performed on a Teledyne ISCO CombiFlash Companion using gradient elution. ^1^H NMR spectra were obtained on Bruker Avance 500, and chemical shifts were determined relative to internal solvent peak.

### Chemistry

The reference compounds **3a**–**3c** were prepared according to our published patent WO 2007/095024.^49^

#### (2-Isopropyl-6-(3-(methylsulfonamido)phenyl)-3-oxoisoindolin-4-yl)boronic Acid (**5a**)

A solution of powdered 4 Å molecular sieves (100 mg, 0.26 mmol), *N*-(3-(7-chloro-2-isopropyl-1-oxoisoindolin-5-yl)phenyl)methanesulfonamide (**4a**, 100 mg, 0.26 mmol), bis(pinacolato)diboron (335 mg, 1.32 mmol), Pd_2_(dba)_3_ (24.2 mg, 0.03 mmol), XPhos (12.6 mg, 0.03 mmol), and KOAc (130 mg, 1.32 mmol) in 2.6 mL of dioxane was heated at 110 °C for 1 h under the atmosphere of argon. Then the reaction mixture was diluted with H_2_O (5 mL) and was extracted with CH_2_Cl_2_ (5 mL × 2). The combined organic layers were dried over MgSO_4_, filtered, and concentrated to give a brown oil. The oil was purified by semipreparative HPLC (30 to 90% gradient). The collected fractions were concentrated and extracted with EtOAc. The combined organic layers were dried over MgSO_4_, filtered, and concentrated and trituration with Et_2_O to afford **5a** (29 mg, yield: 28%) as a white solid. ^1^H NMR (500 MHz, DMSO-*d*_6_) δ ppm: 1.29 (d, 6H), 3.04 (s, 3H), 4.47 (dt, *J* = 13.43, 6.71 Hz, 1H), 4.61 (s, 2H), 7.29 (d, *J* = 7.93 Hz, 1H), 7.38–7.60 (m, 2H), 7.90 (s, 1H), 8.18 (s, 1H), 9.85 (s, 1H).

#### (*S*)-*N*-(3-(1-Oxo-7-(4,4,5,5-tetramethyl-1,3,2-dioxaborolan-2-yl)-2-(1,1,1-trifluoropropan-2-yl)isoindolin-5-yl)phenyl)methanesulfonamide (**5b**)

A solution of (*S*)-*N*-(3-(7-chloro-1-oxo-2-(1,1,1-trifluoropropan-2-yl)isoindolin-5-yl)phenyl)methanesulfonamide (**4b**, 25 mg, 0.58 mmol), bis(pinacolato)diboron (733 mg, 2.89 mmol), Pd_2_(dba)_3_ (21.2 mg, 0.02 mmol), KOAc (170 mg, 1.73 mmol), and XPhos (11.01 mg, 0.02 mmol) in 6 mL of dioxane was heated at 110 °C for 2 h under the atmosphere of argon. The reaction mixture was cooled to rt, filtered through a syringe filter, and washed with EtOAc. The organic solution was concentrated to dryness to afford a brown oil which was purified by preparative HPLC (30 to 65% gradient). The collected fractions were concentrated to and then were basified with NaOH (1 N) to pH 10. The aqueous layer was extracted with CH_2_Cl_2_, and the combined organic layers were dried over MgSO_4_, filtered, and concentrated to afford **5b** (30 mg, yield: 10%) as a white solid. ^1^H NMR (500 MHz, DMSO-*d*_6_) δ ppm: 1.55 (s, 12H), 3.04 (s, 3H), 4.61 (d, *J* = 4.5 Hz, 1H), 4.83 (d, *J* = 4.5 Hz, 1H), 5.11–5.15 (m, 1H), 7.45–7.49 (m, 1H), 7.95 (s, 1H), 8.22 (s, 1H), 9.53 (s, 1H) 9.87 (s, 1H).

#### (2-Cyclobutyl-6-(3-(methylsulfonamido)phenyl)-3-oxoisoindolin-4-yl)boronic Acid (**5c**)

A solution of *N*-(3-(7-chloro-2-cyclobutyl-1-oxoisoindolin-5-yl)phenyl)methanesulfonamide (**4c**, 75 mg, 0.19 mmol), KOAc (56.5 mg, 0.58 mmol), bis(pinacolato)diboron (244 mg, 0.96 mmol), Pd_2_(dba)_3_ (7.03 mg, 7.67 μmol), and XPhos (3.66 mg, 7.67 μmol) in 1.9 mL of dioxane under argon was heated at 110 °C for 4 h. The volatiles were removed, and the residue was purified by preparative HPLC (30–90% gradient). The collected fractions were concentrated under a stream of N_2_ to approximately half volume and were basified by the addition of NaOH (1 N). The resulting aqueous solution was extracted with EtOAc, and the combined organic layers were dried over MgSO_4_, filtered, and concentrated to give **5c** (18 mg, yield: 24%) as a white solid. ^1^H NMR (500 MHz, CDCl_3_) δ ppm: 1.84–1.85 (m, 2H), 2.31–2.34 (m, 4H), 3.06 (s, 3H), 3.80 (s, 1H), 4.59 (s, 2H), 4.90–4.93 (m, 1H), 3.06 (s, 3H), 6.54 (s, 1H), 7.04–7.50 (m, 3H), 7.74 (s, 1H),8.28 (s, 1H).

### Molecular Docking

Computational studies of AZ12559322 were performed with mGluR_2_ homology models developed by Yuan et al.^36^ In brief, the protein model was built using YASARA with the template structures: mGluR1 complexed with glutamate (PDB 1EWK), mGluR5 complexed with glutamate (PDB 3LMK), and apo-form mGluR5 (PDB 6N52). Docking studies were performed with AutoDock Vina (version 1.2.5) and visualized with ChimeraX (version 1.6.1). 2-D protein–ligand interaction plots were generated with Schrödinger Maestro (v 13.8.135).

### Radiochemistry

No-carrier-added [^11^C]CH_4_ was obtained via the ^14^N(p,α) ^11^C nuclear reaction, by irradiating a cyclotron-target (PETtrace 800 cyclotron, GE, Uppsala, Sweden) containing a mixture of nitrogen and hydrogen gas (10%) with a proton beam (16.4 MeV, 35 μA, 10 min). The produced [^11^C]CH_4_ (∼25 GBq) was further converted to [^11^C]CH_3_I via a preestablished gas-phase method. Analytical HPLC was carried out using a gradient pump (L-6200, Hitachi, Tokyo, Japan) and a variable wavelength UV detector (λ = 254 nm, L-4000, Hitachi, Tokyo, Japan) in a series with a Bioscan β+-flow detector. The system was equipped with a reverse phase column (μBondapak, 10 μm, 3.9 × 300 mm) and the products were purified with 40:60 acetonitrile/0.01 M phosphoric acid as the mobile phase, and the flow rate is 3 mL/min. Identification of all radioligands was confirmed by coelution with the corresponding nonradioactive compound. Tritium (^3^H_2_) gas was obtained from RC Tritec (57 Ci/mmol, 2.12 TBq/mmol, 1.06 TBq/matom), and reactions with tritium gas were performed on a TC Tritec manifold.^50^ [^3^H]AZ12229322 was prepared as previously described.^23^ Molar activities were calculated from the mass spectral data using Isopat2.^51^ The assignment of the location of the tritium atoms on [^3^H]AZ12588953 and [^3^H]AZ12592068 was based on the ^3^H NMR of [^3^H]AZ12229322.^23^

### Radiosyntheses of [^11^C]AZ12559322, [^11^C]AZ12592068, and [^11^C]AZ12588953

Prior to the end-of-bombardment, two solutions were prepared: (i) 1,1′-bis(diphenylphosphino)ferrocene-palladium dichloride (2 mg, 2.43 μmol) was dissolved in DMF (250 μL), and (ii) **4a** (4 mg, 10.30 μmol) was dissolved in DMF (50 μL). Both solutions were purged for 20 min with nitrogen gas to remove oxygen. [^11^C]CH_3_I was first trapped at room temperature (RT) in the first solution. Directly following this, potassium orthophosphate (40 μL, 0.04 mmol) was added to the precursor solution, and the reaction mixture was further purged for 3 min. The Pd-complex with the trapped [^11^C]CH_3_I was then transferred to the precursor solution, and the resulting mixture was heated at 100 °C for 4 min by microwave irradiation (CEM Focused Microwave TM Synthesis System). After dilution with the mobile phase, semipreparative HPLC was carried out on a reversed-phase C-18 column (μBondapak, 10 μm, 300 × 7.8 mm) with a UV detector (λ = 254 nm) and a radioactivity detector connected in series. [^11^C]AZ12559322 was purified with a flow rate of 6 mL/min using acetonitrile/0.1% ammonium formate (40:60) as the mobile phase (*t*_R_ = 10 min). The collected fractions were evaporated and redissolved in a mixture of phosphate-buffered saline (10 mL, pH 7.4) and 1 mL of 30% ethanol (in propylene glycol) followed by sterile filtration. Using the present procedure, 920 ± 40 MBq (*n* = 2) of [^11^C]AZ12559322 was obtained in an excellent radiochemical purity (RCP > 98%) and molar activity (*A*_m_ > 37 GBq/μmol). The overall synthesis time for [^11^C]AZ12559322 was 45 min (decay-corrected RCY = 13.4%).

[^11^C]AZ12592068 and [^11^C]AZ12588953 were synthesized according to the radiolabeling procedure of [^11^C]AZ12559322.

### Synthesis of [^3^H]-*N*-[3-(2-Isopropyl-7-methyl-1-oxo-2,3-dihydro-1*H*-isoindol-5-yl)phenyl]methanesulfonamide ([^3^H]AZ12229322)^23^

A flask containing a slurry of 10% Pd/CaCO_3_ (2.1 mg, 1.97 μmol), *N*-(2,4-diiodo-3-(2-isopropyl-7-methyl-1-oxoisoindolin-5-yl)phenyl)methanesulfonamide (1.60 mg, 2.62 μmol), Hunig’s base (10 μL, 0.06 mmol), and absolute ethanol (0.5 mL) was attached to a RC Tritec Tritium Manifold, and the solution was degassed three times using a free-thaw process. The solution was cooled in N_2(l)_, and tritium (342 GBq, 0.160 mmol) was added. The solution was stirred at room temperature for 3 h. The unreacted tritium gas (270 GBq) was recovered, and the remaining volatiles were removed by concentrating the solution to dryness. The residue was taken up in MeOH (1 mL) and concentrated to dryness twice, and the residue was then dissolved in MeOH (1 mL) and filtrated to give a solution containing 2 GBq of tritiated material. The solution was concentrated to dryness, and the residue was purified by preparative HPLC (19 × 250 mm, Waters XBridge C18 5 μ OBD, with gradient elution from 10 to 60% MeCN–0.1% aq TFA over 30 min). Products containing fractions were combined and lyophilized, and the resulting residue was taken up in EtOH (30 mL) to give 1850 MBq. The radiochemical purity of the sample was determined to be 99.3% by HPLC (4.6 × 100 mm Waters XBridge C18 3.5 μm, A: 6.5 mM (NH_4_)_2_CO_3_ adjusted to pH 10, B: MeCN, 0 to 3 min 5% B, 3 to 25 min 5 to 95% B, 25 to 30 min 95% B). ^1^H NMR (CD_3_OD, 500 MHz): 7.29 (m, 1H), 7.46 (m, 2.6H), 7.54 (s, 0.57H),7.61 (s, 1H). ^3^H NMR (CD_3_OD, 550 MHz): 7.46 (d, *J* = 7 Hz, 0.48 T), 7.55 (S, 0.43 T). LC/MS: 357 (63%), 359 (100%), 361 (50%), 363 (5.9%). The specific activity was determined to be 970 GBq/mmol.

### Synthesis of [^3^H]-(*S*)-*N*-(3-(7-Methyl-1-oxo-2-(1,1,1-trifluoropropan-2-yl)isoindolin-5-yl)phenyl)methanesulfonamide([^3^H]AZ12592068)

A slurry of (*S*)-*N*-(4-iodo-3-(7-methyl-1-oxo-2-(1,1,1-trifluoropropan-2-yl)isoindolin-5-yl)phenyl)methanesulfonamide (1.5 mg, 0.003 mmol), 0.92 mg of 10% of Pd/C, and 8 mg of NEt_3_ in 0.5 mL in DMF was degassed three times using freeze thaw. The slurry was cooled in liquid nitrogen, and 590 mCi of ^3^H_2_ was added. The reaction was warmed to rt and was stirred for 4 h. The slurry was cooled in liquid nitrogen, and the residual tritium was recovered. The remaining volatiles were transferred to a waste container, and the residue was taken up in 1 mL of EtOH, which was also vacuum transferred to the waste container (350 mCi total). The residue was diluted with 3 mL of EtOH and was passed through a syringe filter to give 153 mCi of a clear solution in 3 mL of EtOH. A tenth of the crude material was purified by semipreparative HPLC (30 to 70% over 15 min). The combined product containing fractions was diluted with water and passed through a SepPak (Oasis HLB). Elution with EtOH afforded 4.2 mCi in 4.2 mL of EtOH. LCMS (Method A, M + 1): 417 (100%), 415 (68.9%), 413 (9%), 419 (5.5%). Molar Activity: 1591 GBq/mmol. Radio-HPLC (Method C): 99.5%, Radio-HPLC (Method D): 99.5%.

### Synthesis of [[^3^H]]*N*-(3-(2-Cyclobutyl-7-methyl-1-oxoisoindolin-5-yl)phenyl)methanesulfonamide ([^3^H]AZ12588953)

A slurry of *N*-(3-(2-cyclobutyl-7-methyl-1-oxoisoindolin-5-yl)- 4-iodophenyl)methanesulfonamide (1 mg, 0.002 mmol), 1.9 mg of 10% of Pd/C, and 36 mg of NEt_3_ in 0.5 mL in MeOH was degassed three times using freeze thaw methodology. The slurry was cooled in liquid nitrogen, and 400 mCi of ^3^H_2_ was added. The reaction was warmed to rt and stirred for 2 h. The slurry was cooled in liquid nitrogen, and the residual tritium was recovered. The remaining volatiles were transferred to a waste container, and the residue was taken up in 1 mL of MeOH, which was also vacuum transferred to the waste container (300 mCi total). The residue was diluted with 500 μL of EtOH and was passed through a syringe filter to give 55 mCi of a clear solution in 3 mL of EtOH. The compound was purified by semipreparative HPLC in three batches (20 to 60% over 35 min). The combined product containing fractions was diluted with water and passed through a SepPak (Oasis HLB). Elution with EtOH afforded 11.4 mCi of [^3^H]AZ12588953 in 11.4 mL of EtOH. LCMS (Method B): 371 (11.3%), 372 (2.3%), 373 (100%), 374 (21.8%), 375 (27.0%). Molar activity: 1121 GBq/mmol. Radio-HPLC (Method B): 99.5%, Radio-HPLC (Method D): 99.5%.

### In Vitro Autoradiography

The tritium-labeled ligand [^3^H]AZ12559322 for ARG studies was prepared by AstraZeneca.^23^ Briefly, the brain sections of rat or cynomolgus monkey brains in 20 μm thickness were warmed to room temperature in a vacuum chamber over 3 h on the day of the experiment.^52^ All sections were incubated in 50 mM Tris HCl, 5 mM MgCl_2,_ and 0.9% NaCl (pH 7.4) for 5 min at room temperature. The brain sections were dried and incubated with [^3^H]AZ12559322 in the above buffer for 30 min at ambient temperature. For blocking studies, a solution of 5 μM or 10 μM mGluR_2_ PAM (LY-487379: CAS: 353231-17-1; AZD8418: CAS: 1198309-73-7; AZ12522721: CAS: 1092453-89-8) in the incubation buffer was added. Then the tissues were washed in ice cold 50 mM Tris HCl for 3 × 5 min and then dipped twice in distilled water. The tissue sections were dried under fans and exposed to a flashed phosphoimager screen for 5 days. The plates were scanned on a Fuji phosphoimager, and ImageJ was used for image analyses.

### PET Imaging on NHP

PET imaging studies were conducted in one anesthetized healthy cynomolgus monkey and are fully detailed in Dahl et al.^53^ The study was approved by the Stockholm Animal Research Ethical Committee (Dnr. N245/04). The cynomolgus monkey was anesthetized with an intramuscular injection of ketamine hydrochloride (∼10 mg/kg, Ketalar, Pfizer) and maintained by a mixture of sevoflurane (2–8%, Abbott Scandinavia AB), oxygen (∼40%), and medical air after endotracheal intubation. The level of anesthesia was controlled continuously during the experimental session. The head was immobilized with a fixation device, and body temperature was maintained by Bair Hugger model 505 (Arizant Healthcare, MN, USA) and continuously monitored by an esophageal thermometer. ECG, heart rate, blood pressure, respiratory rate, and oxygen saturation were continuously monitored throughout the experiments. Each radioligand was administered ([^11^C]AZ12559322 = 169 MBq; [^11^C]AZ12588953 = 170 MBq; [^11^C]AZ12588953 = 174 MBq) as an intravenous bolus injection during 5 s with simultaneous start of PET data acquisition. Radioactivity in the brain was measured continuously for 123 min using the high-resolution research tomograph (HRRT; Siemens Molecular Imaging, Knoxville, TN, USA) according to a preprogrammed series of 34 frames.

### [^35^S]GTPγS Binding Assay

The in situ GTPγS ARG studies in the rat and NHP brain tissues were conducted according to previous reports.^54^ Briefly, 20 mm thick brain tissue sections were incubated twice in assay buffer, pH 7.5 for 10 min at 25 °C and once in assay buffer with 2 mM GDP for 15 min at 25 °C. Slides were then incubated in an assay buffer containing 0.04 nM [^35^S]GTPgS in the presence or absence of LY379268 and AZD8529 for 2 h at 25 °C. The incubation was stopped by washing twice for 3 min in ice-cold 50 mM Tris–HCl and a quick dip in ice-cold distilled water. Sections were then dried under a cold stream of air and placed in a light-tight cassette with commercially available autoradiography films. Films were developed by standard techniques, digitized, and analyzed.

## Supporting Information Available

Rat in vitro autoradiography studies with [^3^H]AZ125593222 and in situ GTPγS autoradiography of isoindolone derivatives AZD8529 in rat and NHP (PDF)
