# Supplementary material for: Radiosynthesis and Evaluation of 11C-Labeled Isoindolone-Based Positive Allosteric Modulators for Positron Emission Tomography Imaging of Metabotropic Glutamate Receptor 2
Source: ACS Pharmacol Transl Sci. 2024 Jul 10;7(8):2414–23. doi: 10.1021/acsptsci.4c00261 (PMC11320742; doi:10.1021/acsptsci.4c00261)
Supplement: Supplementary file 1 — pt4c00261_si_001.pdf [file pt4c00261_si_001.pdf]

Radiosynthesis and Evaluation of ^{11}C-labeled isoindolone-based positive allosteric modulators for PET imaging of Metabotropic Glutamate Receptor 2

Yinlong Li,[1] Kenneth Dahl,[2, 3] Peter Johnström,[2, 3] Katarina Varnäs,[3] Lars Farde,[3] Christer Halldin,[3] Amy Medd,[4] Donna Maier,[4] Mark E. Powell,[4] Jiahui Chen,[1] Richard Van,[6] Jimmy Patel,[1] Ahmad Chaudhary,[1] Yabiao Gao,[1] Zhendong Song,[1] Ahmed Haider,[1] Yihan Shao,[6] Charles S. Elmore,[4,5] Steven Liang[1,*] and Magnus Schou[2, 3, *]

[1]Department of Radiology and Imaging Sciences, Emory University, 1364 Clifton Road, Atlanta, Georgia 30322, United States.

[2]PET Science Centre, Precision Medicine and Biosamples, Oncology R&D, AstraZeneca, Karolinska Institutet, Stockholm, 17176, Sweden.

[3]Department of Clinical Neuroscience, Centre for Psychiatry Research, Karolinska Institutet and Stockholm County Council, Stockholm, 17176, Sweden.

[4]Neuroscience, BioPharmaceuticals R&D, AstraZeneca, Wilmington, Delaware, 19897, United States.[†]

[5]Early Chemical Development, Pharmaceutical Sciences, R&D, AstraZeneca Pharmaceuticals, Gothenburg 43183, Sweden.

[6]Department of Chemistry and Biochemistry, University of Oklahoma, Norman, Oklahoma 73019-5251, United States.

[†]*The affiliation address provided was valid at the time of data collection but no longer exists.*

Corresponding Authors

Steven Liang − *Department of Radiology and Imaging Sciences, Emory University, 1364 Clifton Road, Atlanta, Georgia 30322, United States.* Email: steven.liang@emory.edu.

Magnus Schou − *PET Science Centre, Precision Medicine and Biosamples, Oncology R&D, AstraZeneca, Karolinska Institutet, Stockholm, 17176, Sweden; Department of Clinical Neuroscience, Centre for Psychiatry Research, Karolinska Institutet and Stockholm County Council, Stockholm, 17176, Sweden*. Email: magnus.schou@astrazeneca.com.

Supporting Information

Content

1) Rat in vitro autoradiography studies with [^{3}H]AZ12559322

2) *In situ* GTPγS autoradiography of isoindolone derivatives AZD8529 in rat and NHP

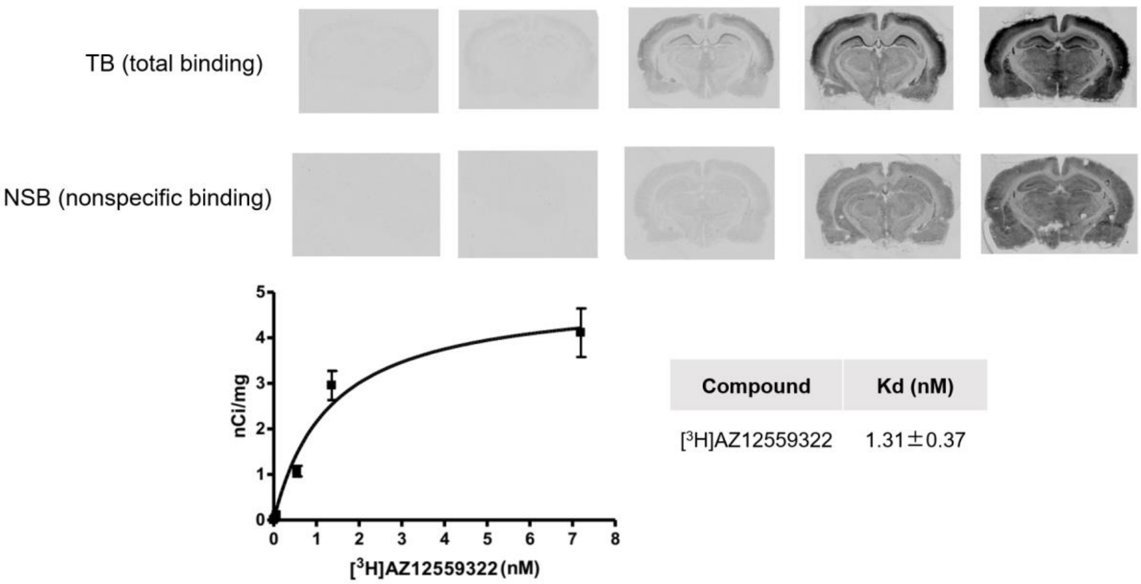

Figure S1. Assessment of [^{3}H]AZ12559322 specific binding by *in vitro* autoradiography studies in rat hippocampus.

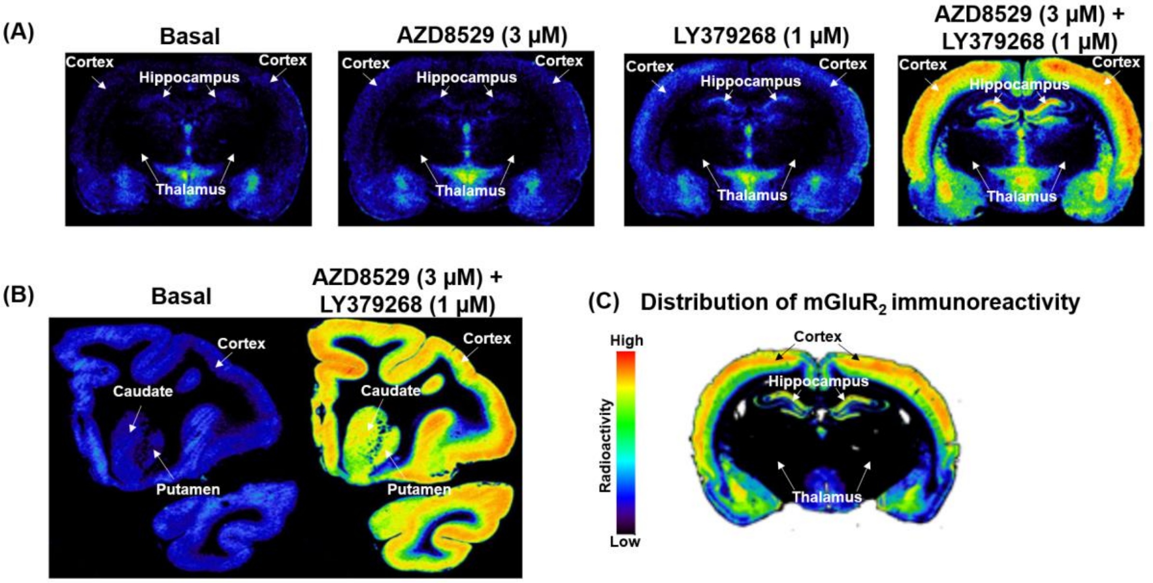

Figure S2. *In situ* GTPγS autoradiography of isoindolone derivatives AZD8529 in rat (A) and NHP brain tissue (B). Immunohistochemical localization of mGluR$_2$ in rat brain tissue (C).
